# Determinants of depressive symptoms among persons 80 years and older: longitudinal national evidence from the health, aging, and retirement study in Thailand, 2015–2022

**DOI:** 10.1186/s12877-024-05479-z

**Published:** 2024-10-26

**Authors:** Supa Pengpid, Karl Peltzer, André Hajek, Dararatt Anantanasuwong, Wasin Kaewchankha

**Affiliations:** 1 Department of Health Education and Behavioral Sciences, Faculty of Public Health, Mahidol University, 420/1 Ratchawithi Road, Ratchathewi, Bangkok, 10400 Thailand; 2 Department of Public Health, Sefako Makgatho Health Sciences University, Pretoria, South Africa; 3 Department of Healthcare Administration, College of Medical and Health Science, Asia University, Taichung, Taiwan; 4 Department of Psychology, University of the Free State, Bloemfontein, South Africa; 5 Department of Psychology, College of Medical and Health Science, Asia University, Taichung, Taiwan; 6 Department of Health Economics and Health Services Research, University Medical Center Hamburg-Eppendorf, Hamburg Center for Health Economics, Hamburg, Germany; 7 Center for Aging Society Research, National Institute of Development Administration, Bangkok, Thailand; 8 Intelligence and Information Center, National Institute of Development Administration, Bangkok, Thailand

**Keywords:** Adults 80 years and older, Depressive symptoms, Longitudinal study, Thailand

## Abstract

**Background:**

Few studies have longitudinally assessed the determinants of depressive symptoms among persons 80 years and older. The aim of this study was to estimate the determinants of depressive symptoms among persons 80 years and older based on 4-wave national longitudinal data from Thailand.

**Methods:**

Data from the Health, Aging, and Retirement in Thailand study from 2015, 2017, 2020 and 2022 were utilized. The sample was restricted to community-dwelling persons 80 years and older (analytic sample: *n* = 2763 observations). For the pooled sample, average age was 85.0 years (range 80–117 years). Established measurements were used to assess depressive symptoms. Linear fixed effects regression was applied to assess the time-variant determinants and outcomes.

**Results:**

Regressions found that higher functional disability and an increase in the number of chronic conditions worsened depressive symptoms. More favourable self-rated physical health, and higher exercise frequency improved depressive symptoms. In addition, among women higher subjective economic status decreased depressive symptoms.

**Conclusions:**

This longitudinal study enhances our understanding of the determinants of depressive symptoms among persons 80 years and older. Strategies to delay or decrease functional disability, chronic conditions, increase physical activity, and improve subjective economic status may help in reducing depressive symptoms.

## Introduction

Thailand’s population is aging quickly [1]. In Thailand, the number of people 80 years of age or older has reached 1.4 million, or 1.9% of the total population. This number is expected to rise quickly at a rate of 7% annually [2] to reach 3 million by 2039 [3]. Increased life expectancy in Thailand has been associated with significant reductions in leading causes of death [4]. However,

functional disability, multimorbidity, and health care needs and costs are probably significantly higher in the 80 + age group than in the younger age groups [5, 6]. Depressive symptoms may be impacted by these social, demographic, and health-related changes [6]. For instance, more depressive symptoms were linked to lower age (i.e., 80–84 years compared to 90 years and over), asset poverty, the presence of multimorbidity, higher functional impairment, and worse self-rated health among adults 80 years of age and older in one German state [6], among the oldest-old in China, financial strain, poor self-rated health, and the presence of heart disease was associated with depression [7], and among persons 85 years and older in Korea, living arrangements and the number of chronic diseases were factors affecting depression [8]. However, these studies on the determinants of depressive symptoms among the oldest-old are cross-sectional, which reduces the ability to establish directionality of the determinants of depressive symptoms [9].

Few studies have longitudinally investigated determinants of depressive symptoms in individuals 80 years and older. For example, in a longitudinal study (2 waves) in one state in Germany found that increases in functional impairment, decreases in self-rated health, decreases in social network size and loss of a spouse worsened depressive symptoms [9]. In an 8-year (2006–2013) cohort study in Korea [10], it was found that among the oldest-old higher family relationship satisfaction, better self-rated health status, higher self-esteem and with time depression significantly decreased, and in a 2-year longitudinal study among the oldest old in Korea, participants who reported a slower increase in positive life events and a faster increase in daily hassles experienced a faster increase in depressive symptoms, while negative life events were not significantly associated with depression [11]. In a longitudinal study among older adults (not oldest old) the prevalence of depressive symptoms significantly increased from before the COVID-19 pandemic in 2018 and 2019 to during the COVID-19 pandemic in June and July 2020, and November and December 2020 [12]. In a cross-sectional study among older adults in Thailand during the COVID-19 pandemic in 2020 found a high prevalence of psychological distress (any positive of a 5-item measure, including symptoms of being sad, unhappy, lonely, no hope in life and loss of appetite) of 51.7% among the oldest-old (which did not significantly differ to younger age groups of 60–69 and 70-79-year-olds) [13].

Given the dearth of information on this subject, especially in relation to longitudinal studies, our study sought to investigate the determinants of depressive symptoms among the very old (80 years and older) (also stratified by sex) based on national, longitudinal community-dwelling data in Thailand from 2015 to 2022. Understanding the elements that contribute to depressive symptoms is essential to developing interventions aimed at assisting the very old.

## Methods

### Participants and procedures

Four waves of health, ageing and retirement in Thailand (HART) studies conducted in Thailand (2015, 2017, 2020 and 2022) were analysed. Through a multi-stage national sampling plan, one adult (over 45 years old) was randomly chosen per household (being the inclusion criterium) and interviewed in the home, with a response rate of 46%; further details are presented elsewhere [14]. In this analysis, the sample was restricted to participants who were 80 years and older. The analytic pooled sample consisted of 2, 763 observations (excluding proxy interviews) in four study assessments in 2015, 2017, 2020, and 2022. From 925 participants (excluding proxy interviews) at baseline, 36 had died, 4 refused and 351 could not be traced. Compared to participants that stayed in the study, participants lost to follow-up did not significantly differ on key sociodemographics (age, education, economic status, living alone, residence and marital status), and health related variables (number of chronic conditions, functional disability and self-rated physical health). Only depressive symptoms were signiticantly lower (*p* < 0.012) in the loss to follow-up group. The Human Research Ethics Committee of the National Institute for Development Administration (ECNIDA 2020/00012) granted approval for the study’s protocol, and participants gave their informed written consent. All methods were carried out in accordance with relevant guidelines and regulations and have been performed in accordance with the Declaration of Helsinki.

## Measures

### Outcome variable

*Depressive symptoms* were assessed with the Center for Epidemiologic Studies Depression Scale (CES-D-10) (total scores 0–30, with higher scores reflecting more depressive symptoms) [15], Cronbach’s alpha was 0.69.

### Independent variables

*Sociodemographic variables* consisted of time varying variables, including age, marital, living and subjective economic status. The latter was evaluated with the question, “How satisfied are you with your economic situation?” (Rated from 0 to 10, with higher scores indicating higher economic status). Time-constant variables included sex, urban-rural residence and educational level, used as sample description, and sex was used in a gender stratified model.

*Religious involvement* was assessed with four items, (1) “Making merit and giving alms according to respected religious principles”, (2) “Prayer in the morning/before going to bed”, (3) “Performing merit-making activities at religious places according to the religions that the interviewees respect on important religious days”, and (4) “Observing important religious days that the interviewees respect.” Response options ranged from 0 = never to 3 = always, total range 0–12, with higher scores indicating greater religious involvement; Cronbach alpha was 0.83.

*Social participation* included participation in at least one of six activities, including social, sports, music, arts and culture, alumni, volunteer and/or political activities. Cronbach’s α was 0.65.

*Frequency of physical exercise or activity* was assessed with the question: “How often do you exercise?” (1 = 0 days, 2 = 1–2 days, 3 = 3–4 days, 4 = 5–6 days or 5 = 7 days per week).

*Self-rated physical health status* was generated from the item, “In general, how would you rate your physical health status?” reported on a “0 (= very poor) to 10 (= excellent)” scale [16].

*Activities of Daily Living (ADL) disability* [17] was defined as not being able to.

dress, wash, eat, or bathe alone (scores 0–4, with higher scores indicating higher functional disability). Cronbach’s α was 0.93.

*Chronic conditions*, included diabetes, hypertension, bone diseases, cardiovascular disease, psychiatric/emotional disorder, brain disease/dementia, liver/gastrointestinal disease, kidney disease, lung disease, prostrate disease, diseases related to the uterus or ovaries, visual impairment, hearing impairment, and cancer are diagnosed by healthcare providers, and self-reported past month urinary incontinence.

### Data analysis

The sample was described using descriptive statistics. Chi-square and t-tests were used to calculate percentage differences for the study waves. In order to estimate the longitudinal relationships between time-varying independent variables and time-varying depressive symtpoms for the four study waves between 2015 and 2022, linear fixed effects (FE) regressions were carried out. The choice of FE regression was confirmed from the Hausman specification test between the fixed-effects model and the random-effects model (for depressive symptoms: (87.76) *p* < 0.001). This model works especially well at mitigating biases resulting from interactions between the individual and factors that are difficult to observe, like genetics; individual fixed effects control for all person-level heterogeneity [18]. Thus, the problem of unobserved heterogeneity is markedly reduced when using FE regressions. Time-varying covariates were included based on earlier review [9–11]. Time-invariant factors, such as sex and education, cannot be included as main effects in linear FE regressions, because they usually do not vary within individuals over time. Therefore, the time-constant factor, sex, was used for stratifying the regression models and education was used for descriptive purposes. Only complete cases (missing cases were < 4% in study variables) were analysed and *p* < 0.05 was considered significant. Cluster-robust standard errors were calculated. StataSE 16.0 (College Station, TX, USA) was used for statistical analysis.

## Results

### Sample characteristics

The pooled analytical sample consisted of 2,763 observations: 925 in wave 1, 723 in wave 2, 581 in wave 3, and 534 in wave 4. For the pooled sample, average age was 85.0 years (range 80–117 years), 43.5% were male, 61.4% were widowed, 14.1% had no formal education, 54.4% were residing in urban areas and 16.3% were living alone. The average level of depressive symptoms was 5.2 (SD = 3.8). Depressive symptoms differed by study wave in the total sample and by age group. Sociodemographic characteristics (age, sex, educational level, and subjective economic status) and covariates (living alone, religious involvement, social participation, exercise, self-rated physical health status, functional disability and sum of chronic conditions) also differed by study wave (see Table 1).

**Table 1:** Analytic sample characteristics, pooled and by study wave, HART 2015–2022

Variable (range)	All waves	Wave 1: 2015	Wave 2: 2017	Wave 3: 2020	Wave 4: 2022	*p*-value^a^
	*N* = 2763	*N* = 925	*N* = 723	*N* = 581	*N* = 534	
	N (%)/Mean (SD)	N (%)/Mean (SD)	N (%)/Mean (SD)	N (%)/Mean (SD)	N (%)/Mean (SD)	
Depressive symptoms (0–30)						
Total sample	5.2 (3.8)	6.8 (3.6)	5.3 (3.8)	3.6 (3.4)	4.2 (3.3)	< 0.001
Age: 80–84 years	5.3 (3.8)	6.5 (3.5)	5.4 (3.9)	3.7 (3.6)	4.2 (3.6)	< 0.001
Age: 85–89 years	5.1 (3.7)	6.9 (3.6)	5.0 (3.7)	3.6 (3.1)	4.1 (3.2)	< 0.001
Age: 90 or more years	5.3 (3.9)	7.5 (3.9)	5.6 (3.9)	3.2 (3.5)	4.3 (2.9)	< 0.001
Age (80–117 years)	85.0 (4.3)	84.5 (4.1)	85.2 (4.3)	85.3 (4.1)	85.5 (4.5)	< 0.001
Sex (male)	1274 (43.5)	442 (47.8)	326 (44.5)	240 (40.0)	266 (39.7)	0.003
Marital status						
Single/divorced/separated/married	1056 (38.6)	347 (38.4)	297 (41.1)	219 (37.8)	193 (36.3)	0.325
Widowed	1682 (61.4)	556 (61.6)	426 (58.9)	361 (62.2)	339 (63.7)	
Education						
None	411 (14.1)	156 (16.9)	111 (15.3)	76 (12.6)	68 (10.2)	< 0.001
Primary	2306 (79.2)	715 (77.3)	575 (79.2)	476 (80.1)	540 (81.0)	
> Primary	195 (6.7)	54 (5.8)	40 (5.5)	42 (7.1)	59 (8.8)	
Subjective economic status (0–10)	6.3 (2.1)	6.2 (2.0)	6.1 (2.2)	6.1 (2.2)	6.9 (1.9)	< 0.001
Residence (urban)	1592 (54.4)	527 (57.0)	383 (52.3)	310 (51.7)	372 (55.5)	0.113
Living alone	476 (16.3)	110 (11.9)	185 (25.2)	98 (16.3)	83 (12.6)	< 0.001
Religious involvement (0–12)	6.2 (3.6)	6.0 (3.6)	6.3 (3.6)	5.9 (3.6)	6.8 (3.8)	< 0.011
Social participation	839 (28.7)	254 (27.5)	227 (31.0)	190 (31.7)	168 (25.1)	0.024
Exercise frequency (1–5)	2.4 (1.7)	2.3 (1.7)	2.7 (1.8)	2.3 (1.7)	2.3 (1.7)	< 0.001
Self-rated physical health (0–10)	6.7 (1.8)	6.4 (1.9)	6.3 (1.9)	7.0 (1.7)	7.3 (1.6)	< 0.001
Functional disability (0–4)	0.3 (0.9)	0.3 (0.9)	0.3 (0.9)	0.5 (1.1)	0.4 (0.9)	< 0.001
Number of chronic conditions (0–15)	1.7 (1.4)	1.3 (1.2)	1.6 (1.3)	1.7 (1.3)	2.3 (1.5)	< 0.001

^a^based on Chi-square tests or t-tests

Table 2 shows the sample characteristics by sex. Depressive symptoms did not differ by sex in the total sample and by age group. Oldest-old women were more likely widowed, had lower education, lower subjective economic status, lower social particiption and lower exercise frequency than men (see Table 2).

**Table 2:** Analytic sample characteristics by sex, pooled over four study waves, HART 2015–2022

Variable (range)	Male	Female	*p*-value^a^
	*N* = 1219	*N* = 1544	
	N (%)/Mean (SD)	N (%)/Mean (SD)	
Depressive symptoms (0–30)			
Total sample	5.17 (3.8)	5.24 (3.8)	0.631
Age: 80–84 years	5.23 (3.8)	5.29 (3.8)	0.778
Age: 85–89 years	5.08 (3.8)	5.06 (3.6)	0.943
Age: 90 or more years	5.14 (3.7)	5.46 (4.1)	0.415
Age (80–117 years)	84.9 (4.1)	85.1 (4.3)	0.179
Marital status			
Single/divorced/separated/married	745 (61.5)	311 (20.4)	< 0.001
Widowed	467 (38.5)	1215 (79.6)	
Education			
None	105 (8.7)	293 (19.1)	< 0.001
Primary	987 (81.4)	1191 (77.5)	
> Primary	120 (9.9)	53 (3.4)	
Subjective economic status (0–10)	6.4 (2.1)	6.2 (2.1)	0.010
Residence (urban)	671 (55.0)	815 (52.8)	0.237
Living alone	195 (16.1)	256 (16.6)	0.713
Religious involvement (0–12)	6.1 (3.7)	6.3 (3.5)	0.067
Social participation	384 (31.5)	392 (25.4)	< 0.001
Exercise frequency (1–5)	2.55 (1.8)	2.25 (1.7)	< 0.001
Self-rated physical health (0–10)	6.73 (1.8)	6.61 (1.9)	0.078
Functional disability (0–4)	0.31 (0.9)	0.35 (1.0)	0.211
Number of chronic conditions (0–15)	1.63 (1.4)	1.72 (1.4)	0.100

^a^based on Chi-square tests or t-tests

### Determinants of depressive symptoms

The findings of the linear FE regression demonstrated a significant correlation between the reduction of depressive symptoms and increasing age in the overall sample (β -0.25, *p* < 0.001), in men (β -0.26, *p* < 0.001), and in women (β -0.24, *p* < 0.001). Furthermore, decreases in depressive symptoms were significantly associated with better subjective economic status among women (*β* -0.12, *p* = 0.013). In addition, decreases in depressive symptoms were correlated with increases in physical activity or exercises in the total sample (*β* -0.20, *p* < 0.001), among men (*β* -0.19, *p* = 0.043), and among women (*β* -0.20, *p* = 0.016). Decreases in depressive symptoms were correlated with increases in self-rated physical health status in the entire sample (*β* -0.21, *p* < 0.001), among men (*β* -0.23, *p* = 0.015), and among women (*β* -0.21, *p* = 0.004). More depressive symptoms were correlated with a higher number of chronic conditions in the entire sample (*β* 0.20, *p* = 0.024), and among women (*β* 0.26, *p* = 0.028), and increases in depressive symptoms were significantly associated with increases in the number of ADL disabilities in the entire sample (*β* -0.24, *p* = 0.038), and among men (*β* 0.49, *p* = 0.010) (see Table 3).

**Table 3:** Determinants of depressive symptoms from fixed-effects regression analyses (waves 1, 2, 3 and wave 4)

Independent variables	All		Male		Female	
		p-value	UBC (RSE)	p-value	UBC (RSE)	p-value
Age	-0.25 (0.03)	< 0.001	-0.26 (0.06)	< 0.001	-0.24 (0.05)	< 0.001
Marital status						
Single/divorced/separated/married	(Reference)		(Reference)		(Reference)	
Widowed	-0.25 (0.36)	0.431	-0.74 (0.54)	0.170	-0.04 (0.47)	0.938
Subjective economic status	-0.08 (0.05)	0.084	-0.03 (0.07)	0.631	-0.12 (0.06)	0.013
Living arrangement						
Living with others	(Reference)		(Reference)		(Reference)	
Living alone	-0.11 (0.28)	0.632	-0.26 (0.44)	0.558	-0.02 (0.35)	0.943
Religious involvement	0.01 (0.03)	0.694	0.02 (0.04)	0.652	0.002 (0.3)	0.950
Social participation	-0.14 (0.28)	0.632	0.13 (0.37)	0.721	-0.32 (0.35)	0.360
Exercise frequency	-0.20 (0.06)	< 0.001	-0.19 (0.09)	0.043	-0.20 (0.08)	0.016
Self-rated physical health	-0.21 (0.06)	< 0.001	-0.23 (0.09)	0.015	-0.21 (0.07)	0.004
Functional disability	0.24 (0.11)	0.038	0.49 (0.19)	0.010	0.07 (0.17)	0.699
Number of chronic conditions	0.20 (0.09)	0.024	0.14 (0.12)	0.232	0.26 (0.12)	0.028
Constant	28.59 (3.39)	< 0.001	29.03 (4.87)	< 0.001	27.26 (4.54)	< 0.0012
Observations	2709		1197		1512	
Individuals	1488		675		825	
R^2^	0.03		0.03		0.02	

UBC = Unstandardised Beta Coefficient; RSE = Robust Standard Errors

The main model was expanded by including year (period) effects in a sensitivity analysis. However, in terms of effect sizes and significance, our main findings stayed essentially unchanged. The final step involved adding interaction terms to the main model for both sex and independent variables. The interaction terms did not, however, become statistically significant.

## Discussion

The aim of this study was to estimate for the first time the determinants of depressive symptoms among the very old (80 years and older) in Thailand based on national, longitudinal community dwelling data from 2015 to 2022. FE Regressions showed that an increase in the number of chronic conditions increased depressive symptoms, and increasing age, higher self-rated physical health and higher exercise frequency decreased depressive symptoms. In addition, among women, higher subjective economic status decreased depressive symptoms. An increase in functional disability increased depressive symptoms in the total sample and among men but not women, showing the vulnerability of men developing depressive symptoms because of functional disability.

In terms of age, the study showed that depressive symptoms decreased with age. In a cross-sectional study in Thailand the prevalence of psychological distress did not significantly differ between the oldest-old and younger age groups of 60–69 and 70–79 year-olds [13]. Similarly to our findings, in two longitudinal studies among the oldest-old in Korea [10, 19] and one in Sweden [20], depressive symptoms declined over time. One possible explanation for this could be related to selective attrition [20], but in our study those who were lost to followp up had a lower depression score than those who stayed in the study, and those who died also had a lower depression score (3.8) than those who stayed in the study (5.2). Another possible explanation is that resilience may increase with age, leading to lower depressive symptoms [21]. For example, individuals of the oldest-old with higher age may have already overcome major negative life events, such as becoming a widow or widower, than the younger oldest-old and their levels of depressive symptoms bounces back to baseline levels [6]. A previous longitudinal study among older adults (not oldest old) in England found a significant increase in the prevalence of depressive symptoms from before the COVID-19 pandemic in 2018 and 2019 to during the COVID-19 pandemic in June and July 2020, and November and December 2020 [12], while this study found a significant decrease of depressive symptoms from prior to COVID-19 pandemic assessments in 2015 and 2017 to assessments during the COVID-19 pandemic in 2020 and 2022.

Consistent with previous longitudinal studies including the very old [9, 10], we found that increasing functional impairment in the total sample and among men, an increase in the number of chronic conditions overall and among women and decreasing self-rated physical health status overall, among men and among women worsens depressive symptoms. Reasons for the gender-gap include that according to a recent review [22] older women may have a higher number of chronic conditions, suffer from poorer general health, and are more likely to be widowed than older men. In this study the oldest-old adult women were more likely widowed than men, but there were no significant sex differences in the number of chronic conditions, and self-rated physical health as well as functional disability. Having chronic conditions may have detrimental effects on different body organs, resulting in feelings of disempowerment and negative emotions that may lead to depression [23]. The association between increasing self-rated physical health and decreasing depressive symptoms may be explained by self-rated physical health not declining with age to the same extent as chronic diseases and functional disability increase [24]. Furthermore, the associations between functional disability, multimorbidity and depressive symptoms may also be bidirectional, as found in a previous study showing a bidirectional association between multimorbidity and depressive symptoms among older adults in Thailand [25], and bidirectional relationship between functional limitations and depressive symptoms among centenarian survivors in their 80s in USA [26].

Furthermore, in line with previous cross-sectional findings [6, 7, 27], we found that higher subjective economic status among women and higher exercise frequency overall and among men and women, decreased depressive symptoms. This may show the special vulnerability of poorer economic status among the oldest-old women, as in this study older women rated their subjective economic status as lower than in older men. Economic strain may weaken sense of control leading to increased depressive symptoms [28]. To address older people’s income insecurity, the Thai government may wish to expand its “pension coverage and benefits.” [29]. Studies have demonstrated [30, 31] that physical activity enhances mental health and reduces depressive symptoms. Possible mechanisms involved in exercise or physical activity improving mental well-being could be a reduction of inflammatory markers implicated in chronic disease, depression, and disability [32]. Considerung the physical functioning of the very old, physical activity should be promoted, possibly with the help of relatives and/or health volunteers. A systematic review of exercise interventions among the oldest-old showed that single-component and multicomponent exercise interventions produced positive effects on muscle strength and balance [33]. Older adults with better socioeconomic status may utilize the tools available to them so they can adopt more healthful behaviours, such as physical exercise [34], and thus reduce depressive symptoms.

Unlike some previous research [9], we did not find that increases in widowhood, decreases in social participation and religious involvement increased depressive symptoms. It is possible that no significant effect of religious involvement was found because overall levels of religious involvement were high (6.2).

### Study strength and limitations

The study includes a longitudinal, national community-dwelling sample and established measures from the HRS and KLOSA, such as the CES-D. One study limitation is that all data were assessed by self-report, and future assessments should also include objective measures. Some factors that have been demonstrated to affect depressive symptoms, like dietary habits [35, 36], cognitive impairment [37], stressful life events [11] and childhood adversity [38] were not evaluated and ought to be taken into account in subsequent studies (when data are available). Furthermore, the HART study suffered from a high loss of follow-up and has a relatively short follow-up period (usually two years), which limits the evaluation of long-term development of depressive symptoms. A low response rate may increase the likelihood of a sampling bias. However, attrition analysis in this study showed no significant differences in terms of sociodemographic, social and health variables. Finally, the sample did not include oldest-old adults living in institutional or assisted living communities, which may have yielded higher rates of depression than in community dwelling oldest-old adults [39].

## Conclusion

This longitudinal study markedly enhances our understanding of the determinants of depressive symptoms among persons 80 years and older in Thailand. Strategies to delay or decrease functional disability, chronic conditions, increase physical activity, and improve subjective economic status may help in reducing depressive symptoms. In addition to the current policies, separate programmes for the oldest-old should be developed and implemented, such as physical activity promotion, screening and management of chronic conditions, and financial interventions. Future research should include more comprehensive measures, including dietary habits, cognitive impairment, stressful life events and childhood adversity, and longer successful follow-up periods.

## Data Availability

Data is publicly available at Health, Aging, and Retirement in Thailand (HART): https://hart.nida.ac.th/download-center/.
